# Chunks, pauses, and holistic processing in Mandarin spontaneous speech

**DOI:** 10.3389/fpsyg.2023.1071729

**Published:** 2023-02-16

**Authors:** Dongyue Xie, Hua Chen, Bin Li

**Affiliations:** ^1^School of Foreign Studies, Nanjing University, Nanjing, China; ^2^Department of Linguistics and Translation, City University of Hong Kong, Hong Kong, China; ^3^Department of Applied Foreign Language Studies, Nanjing University, Nanjing, China

**Keywords:** chunk, pause, holistic processing, phonological coherence, spontaneous speech

## Abstract

Chunks are multiword sequences with independent meaning and function, or formulaic based on the intuition of native speakers, hypothesized to be holistically restored and retrieved in the mental lexicon. Previous studies suggest that pauses and intonational boundaries tend to occur at the boundaries of chunks, but less discussion was made on the influence of chunk categories over mental processing and on pause placement associated with intonational continuity. This study adopted spontaneous monologs of Mandarin natives in formal and informal settings. It examined the co-occurrence of chunks and pause-defined processing units and pause placement around chunks to explore to what extent chunks are holistically processed. The results showed that Mandarin chunks were likely to be situated within a single processing unit, indicating chunks as smaller units than processing units in spontaneous speech. Major chunk categories exhibited significantly different patterns in co-occurring with processing units, indicating the influence of chunk properties on the mental processing of chunks. In addition, chunks tended to be fluently processed in spontaneous speech production as fewer hesitations occurred before and during chunk production. Major chunk categories shared a similar threshold in encountering hesitations before chunk production and differed significantly in hesitation distribution during chunk production. Hesitations in the middle of chunks were more likely to be situated within intonation units compared to those before chunk production. Speakers’ effort to maintain the intonational continuity of chunks when they encounter processing difficulties reveals the mental reality of the holistic nature of chunks. Furthermore, the co-occurrence of chunks and processing units differed significantly between the formal and informal speech genres, indicating genre influence on the mental processing of chunks. Altogether, the findings of this study have provided implications for theories on chunks and the syntactic-prosody interface and contributed to implications for the design of Mandarin instructions and teaching.

## 1. Introduction

The phenomenon of chunking has been studied under various terminologies across multiple disciplines, such as psycholinguistics, corpus linguistics, and second language acquisition. They are of various categories regarding their structural and functional properties, such as collocations, frame constructions, idioms, conversational conventions, etc. Chunks profoundly impact language acquisition (Myles and Cordier, 2017) and are crucial to speech communication and language use (Hallin and Van Lancker Sidtis, 2015). One influencing proposal on the underlying psycholinguistic mechanism of chunks hypothesizes that chunks are “stored and retrieved whole from memory” and do not subject to grammatical analysis (Wray, 2002, p. 9). Over recent decades, this proposal has gained empirical support as researchers identified mental processing advantages of chunks over novel phrases through psycholinguistic experiments (*Cf.* Schmitt and Underwood, 2004; Jiang and Nekrasova, 2007). However, refutes against these findings are also raised, arguing that holistic storage and retrieval cannot be equalized to faster reaction speed (Siyanova-Chanturia, 2015). Meanwhile, pauses and prosodic cues, such as intonational boundaries, are proposed as indirect indicators of mental processing (Warren, 2016) and contribute to detecting formulaicity (Hickey, 1993; Wray, 2002; Lin, 2018). Researchers have proposed that chunks exhibit the property of phonological coherence based on language acquisition observations (Peters, 1983; Wood, 2006). Previous findings suggest that a chunk tends to occupy a single intonational unit (Lin and Adolphs, 2009; Lin, 2018) and is less likely to encounter pauses compared to non-prefabricated strings (Erman, 2007; Schneider, 2014). However, few studies associate pauses with speakers’ intonation performance since there could be hesitations embedded within intonation units (Lin, 2018). Moreover, pause types around chunks are underexplored, and the relationship between chunk categories and pause placement remains to be discussed, as different chunk categories may undergo different mental processes (Carrol and Conklin, 2019).

Moreover, chunks also exist in the Mandarin language such as idiomatic expressions, such as “爱面子 (be concerned about one’s face-saving)” and sentence builders such as “不但…而且…(not only… but also…),” and they are also suggested to bear the holistic nature (Qian, 2008; Wang, 2013). Meanwhile, as a tone language, Mandarin shares a different prosodic system compared to English in several ways, but people using Mandarin do make prosodic segmentation and hesitations in speech flow (Tao, 1996). Therefore, it is worth investigating the prosodic realization of Mandarin chunks, which would project further discussions on the holisticity of the mental processing of chunks.

Based on the above research background, this study explores and analyzes how chunks co-occur with processing units delineated by pauses and the influence of chunk category on pause placement before and in the middle of chunks. It also discusses the relationship between hesitation placement around chunks and intonation units. In addition, the influence of speech genre on the mental processing of chunks has also been discussed. The findings of this study would provide empirical evidence on how chunks are processed prosodically and reveal the mental processes during chunk production in Mandarin spontaneous production. Moreover, exploration of the prosodic manifestation of Mandarin chunks would further our knowledge of the mental processing mechanism of chunks from a different language other than English.

## 2. Review of literature

### 2.1. Chunks and holistic processing

The phenomenon of chunking has been studied under various terms which emphasize the linguistic properties of chunked units from different perspectives. Among the many research attempts, chunks are regarded as shared knowledge among speaker groups (Erman and Warren, 2000; Foster, 2001), consisting of at least two words and bearing an independent meaning or function (Wray, 2002; Wood, 2015). Acknowledging the consensus of chunks and the critical role of characters in the Mandarin language (Wang, 2013), this study defines a Mandarin *chunk* as a sequence of at least two words (a Mandarin word could be one character) with an independent meaning and formulaic based on the language intuition of native speakers. Previous researchers have proposed different chunk categories according to the structural and formal properties of chunks, including fixedness, continuity, grammatical level, and meaning transparency (Nattinger and DeCarrico, 1992; Erman and Warren, 2000; Wang, 2013).

The idea that chunks would exhibit prosodic features is based on two assumptions that a chunk is holistically restored and retrieved in our mental lexicon and that prosodic cues reveal the mental processing of a speaker (Lin, 2018). Wray (2002, p. 9) proposed that chunks are prefabricated, retrieved whole from memory, and not “subject to generation or analysis by the language grammar.” Wray (2002) interpreted the proposal through the dual system of analytical and holistic processing and argued that chunks were holistically processed as many chunks either fail grammatical explanation or offer a limited range of forms and meanings. Moreover, Wray’s proposal on the prefabrication of chunks falls into the assumption proposed by the ACT (Adaptive Control of Thought) theory (Anderson, 1983), which claims that prefabricated multiword units are restored in declarative memory and activated by the route of spreading activation. Similarly, the usage-based exemplar model explains the holistic nature of chunks as the consequence of repeated exposure to the linguistic phenomenon and postulates that sequences are stored as wholes in memory from the first encounter (Bybee, 2010). Both the ACT theory and the exemplar model associate prefabrication of chunks with less processing effort and faster processing speed and have received support from psycholinguistic attempts, such as eye-tracking (*cf.* Underwood et al., 2004) or self-paced reading (*cf.* Kim and Kim, 2012) studies. However, Siyanova-Chanturia (2015, p. 13) argued that more empirical research on the “activation, prominence or modifiability” of chunks was needed to address the issue of holistic storage and processing rather than only on processing speed. In addition, the hypothesis of holistic storage and retrieval is yet to explain the mental processing of chunks by second language learners. As Bardovi-Harlig (2009) observed, there is a mismatch between acquired formulas and the formulas in actual use, indicating the chunks holistic restored could be unsuccessfully retrieved.

Previous studies on Mandarin chunks suggest a similar property of holistic storage and retrieval (Wang, 2013; Kong, 2018). Existing studies support processing advantage for idioms (Yu et al., 2016), N-grams (Kong et al., 2016), and collocations (Jiang, 2021) over novel language by native speakers due to factors including decomposability, familiarity, and structural properties. Despite psycholinguistic attempts at chunk production in labs, less exploration is made into the realization of Mandarin chunks in spontaneous speech production.

### 2.2. Chunks and pauses

Pauses and prosodic cues are important indicators for speech planning and leave traces of syntactic organizing and lexical searching behavior of a speaker (Goldman-Eisler, 1968; Rochester, 1973; Chafe, 1994). The significant role of pauses in spontaneous speech is manifested through pause placement and pause types and is found to correlate syntactic structures and phonemic clauses. Predominant planning points are at the sentence and phrase boundaries (Clark and Clark, 1977) and the boundaries of intonation units (Boomer, 1965; Tree and Clark, 1997). In addition, previous studies differentiated *grammatical pauses* (or juncture pauses) for grammatical and communicative junctures and *hesitation*s [or production pauses in Erman (2007)] that bear unexpected cognitive difficulties of a speaker. Hesitation phenomena, such as filled and unfilled pauses, drawls, speech repairs, and false starts, are regarded as indicators of the chunkiness of word sequences (Bybee, 2007; Schneider, 2014).

Fluent, non-hesitant production and one of the essential characteristics of chunks in spontaneous speech production (Bardovi-Harlig, 2009). Existing findings support that speakers tend not to interrupt mentally coherent units through hesitations (Goldman-Eisler, 1968; Beattie and Butterworth, 1979; Krivokapić, 2012). Chunks, assumed to be holistically restored and retrieved, are suggested to bear the property of phonological coherence, as they tend to be fluently retrieved and produced, unlikely to encounter hesitations ahead and internally (Wood, 2006; Lin, 2018). Pauses or hesitation phenomena often take place at chunk boundaries based on the observation of child language (Peters, 1983) and the speech of foreign language learners (Dechert, 1983; Raupach, 1984; Weinert, 1995). Studies show that word strings with stronger internal bonds are less likely to encounter internal pauses (Bybee, 2007). Erman (2007) found fewer production pauses in manually identified prefabs (11.3%) than in non-prefabricated strings (88.7%) in the COLT and LLC.^1^ In addition, she also identified that the cognitive fluency of chunk production differed significantly between the adolescent and adult speaker groups, indicating the mental processing of chunks could be stylistically different. Schneider (2014) investigated the correlation between hesitation placement and two-word sequence in the Switchboard NXT corpus^2^ and found that hesitation markers were significantly less within two-word collocations of high mutual information value and frequency. Hesitation markers were more frequently found at phrasal boundaries and before content words. The number of hesitations differed along with the complexity of verb clauses and the number of additional segments before the subject.

### 2.3. Chunks and processing units

The processing unit in spontaneous speech has been discussed from the perspectives. Grosz and Sidner (1986, p. 177), from a discourse structuring perspective, regarded processing units as “the sequence of utterances,” while Frederiksen (1977) and Hobbs (1978) interpreted processing units according to propositional properties and logical relations. Moreover, Ford and Holmes (1978, p. 35) proposed that major “planning units” in sentence production are the deep clauses based on their observation of speakers’ prosodic behavior. Through different proposals, a common practice to trace speech planning and processing would be through pauses and hesitations (Boomer, 1965; Butterworth, 1975). Mental processing is covertly practiced by language users in spontaneous speech production, and pauses and hesitations leave traces of the undergoing syntactic organizing and lexical searching behaviors of a speaker. Therefore, this study defines *processing units* as a word sequence divided by pauses or a “pause-defined unit” (Dechert, 1983; Brown et al., 2015).

As previously reviewed, researchers speculated that chunks tend to be delineated by pauses due to their holistic nature (Dechert, 1983; Raupach, 1984; Weinert, 1995). Research attempts are made to evaluate whether pauses are reliable indicators of chunk boundaries, as Wray (2002, p. 37) predicted that the patterns of pause placement around chunks would be “unprincipled” due to the fixedness of chunk frames. Dahlmann and Adolphs (2007) found that pauses did not always occur at the boundaries of highly frequent 3-word n-grams “I do not know” and “I think I,” and the former chunk has fewer internal pauses, indicating holistic storage to chunks with a holistic meaning. In addition, Lin (2018) found that 82.26% (51 out of 62) of the formulaic sequences identified by native speaker judgment tasks in the NMMC^3^ were not interrupted by pauses, and only 9 out of 62 formulaic sequences were delineated by pauses. She argued that chunks are more likely to be marked by intonation boundaries rather than pauses, as previous researchers speculate that chunks often form a single intonation unit due to their fixedness and lexicalization (*cf.* Altenberg and Eeg-Olofsson, 1990; Aijmer, 2014). Moreover, Lin and Adolphs (2009) proposed four possible alignment situations between chunks and intonation units and examined the most frequent 5-word sequence, “I do not know why,” and its intonational boundaries in non-native English conversations from the NICLEs-CHN^4^. Among the 56 cases identified, 55% occupy a single intonation unit, and 85% align with at least one side of the intonation unit boundaries. In the follow-up studies, Lin (2010, 2018) identified 62 chunks in native adult lecture speech from NMMC through native speaker identification tasks and found that 40.3% took up an independent intonation unit. Chunks that aligned with one side of intonation units made up 82.3%. Lin’s (2010, 2018) findings are consistent with Lin and Adolphs (2009) and support the claims that chunks often occupy one intonation unit. Lin (2010, 2018) also suggested genre differences for such co-occurrence due to the variations identified in the series of studies. However, Lin and Adolphs (2009) and Lin (2018) did not consider internal speech dysfluency and hesitation phenomena in intonation units.

So far, previous studies have suggested that chunks tend to be holistically restored and retrieved and unlikely to be interrupted by pauses. It is also assumed that if chunks are holistically stored and retrieved, then there would be prosodic indicators to reflect such a processing mechanism. However, it remains to be explored to what extent chunks would be marked by pauses and form independent processing units in spontaneous speech. In addition, fewer discussions have been made on the influence of chunk categories on the mental processing of chunks, as previous attempts either studied particular chunks or evaluated chunks of different types as a whole. Moreover, previous studies on pause placement around chunks did not differentiate pauses for grammatical and function junctures and hesitations, which would further the understanding of how chunks are processed in spontaneous speech. Despite the tendency of chunks to be situated within intonation units, further investigations are needed to assess the relationship between hesitations around chunks and intonation units, as hesitation phenomena would occur within intonation units. As previous studies have suggested, speech genre and speaker group may influence the mental processing or prosodic package of chunks. On the one hand, the nature of different speech genres may exert different degrees of psychological pressure on the working memory of a certain speaker. For instance, Fillmore (1979) observed that sports commentators fill time by producing utterances at length with few pauses and hesitations without having time to consider what to say next. Other the other hand, chunks can contribute to or enhance the style of a particular speech genre (Wray, 2002). Some chunks could be more heavily used than other discourse contexts (Oakey, 2010). It thus requires a more inclusive view of chunk processing by incorporating more speech genres as different speech genres may contain a different proportion of chunks (Biber, 2004).”

Therefore, the current study proposes the following three research questions:

1. Are chunks always delineated by pauses in Mandarin spontaneous speech? If not, how do chunks of different categories co-occur with processing units?
2. Are chunks fluently processed in Mandarin spontaneous speech? If not, how are hesitations around chunks situated in intonation units?
3. Do chunks co-occur with processing units similarly across speech genres? What characterizes patterns or variations across speech genres?

## 3. Materials and methods

### 3.1. Data and prosodic annotation

The current study selected monologs by Mandarin adult natives in two speech settings, formal and informal. Each contains an effective length of speech of 1 h and 20 min. The formal and informal speech data differ regarding different degrees of speech formality and discourse topics. The formal speech setting^5^ (16.3 thousand Mandarin characters) includes political commentary by researchers on political research through television broadcasts. In contrast, the informal setting^6^ (15.8 thousand Mandarin characters) consisted of host speeches by well-trained hosts and hostesses, containing daily expressions, anecdotes, and jokes at the closing ceremonies of independent film festivals. All video clips were converted into wav. Format and transcribed manually.

The audio data was annotated *via* Praat (6.2.03) by professional phoneticians on Mandarin phonetics who were naïve about the research objectives. The annotation follows the external and internal criteria proposed by Cruttenden (1997, pp. 29–36). The external criteria are prosodic indicators, including pauses above 200 ms (Raupach, 1984; Schneider, 2014), pitch reset, final syllable lengthening, and anacrusis. The internal criteria require an intonation unit to contain one nuclear and bear pitch movement.

### 3.2. Identification of processing units

In the current study, processing units are word sequences delineated by both grammatical pauses and hesitations. Grammatical pauses are any perceivable silent pauses between clauses that contribute to long-term grammatical and semantic planning and facilitate the intelligibility of speech (Reich, 1980, p. 380). Comparatively, hesitations concern a variety of dysfluency phenomena, including filled and silent pauses, drawls (syllable lengthening), repetition, and self-repair. Silent pauses for hesitation differ from grammatical pauses by their placement in utterance as they are located at points of low transitional probability within clauses. In order to avoid over-exploitation of silent pauses in the data, silent pauses for hesitation were holistically perceived and identified according to the following criteria: (1) A silent pause for hesitation often occurs at the lower nodes of a syntactic structure. (2) if a pause before words exceeds or is close to the pause length at phrase boundaries nearby, it would be recognized as a marker of hesitation. (3) Silent pauses for hesitation are often accompanied by additional hesitation phenomena, such as filled pauses, drawls, or unnatural pronunciation. In addition, four main filled pauses in Mandarin, including “呃,” “嗯,” “这个,” and “那个,” were identified. Lexical fillers, “这个” and “那个,” were regarded as filled pauses only when they lost their referential meaning in discourse.

### 3.3. Identification of chunks

Chunks in this study were identified through the native speaker judgment task, which aims to locate chunks that best fit the linguistic intuition of the native speakers. Previous studies have preferred the use of external judges other than researchers themselves to avoid circular arguments and enhance the validity of identification results in previous studies (Erman and Warren, 2000; Foster, 2001; Wood, 2006; Lin, 2018), and the number of judges ranges from 2 to 30 according to the size of the dataset. Judges in these studies were either linguistic experts or laypeople and identified chunks according to multiple hints and identification criteria. Wulff (2008) suggested that the ability to make formulaicity judgments is shared by both linguistic professionals and laypeople. However, linguistic professionals remain a proficient option for a heavy identification task.

This study invited six adult Mandarin natives as judges for the identification task who were innocent of the research objectives and the speech material. They firstly received a training session by the author, which explained the definition of chunks in Mandarin, identification criteria, and taxonomies with limited examples from literature. The definition of chunks was described to the judges as: “A chunk is a combination of at least Mandarin words (a Mandarin word could be one character only) that has a metaphorical or pragmatic meaning, or it is extremely common of language use. A chunk can also be a frame of multi-word as a phrase or sentence stem.”

The criteria for chunk identification were grounded in Wray’s (2008) proposal, which encompassed a package of chunk properties from multiple perspectives. The study deliberately removed the phonological criteria to avoid circular issues. In addition, the taxonomies in this study adopted previous proposals for the major and subordinate categorization of Mandarin chunks (Wang, 2013; Lin, 2018). Table 1 shows major chunk categories, including collocations, frame constructions, and institutionalized expression. Collocations are content word combinations of common usage and differ in the degree of fixedness. They include fixed collocations and collocations with restricted lexical choices for combination, transparent in meaning (Qian, 2008). Modified collocations, as compared to direct collocations, allow lexical insertions (Jiang, 2021), for instance, “引起强烈的共鸣 (arouse strong resonance).” Comparatively, frame constructions often involve prepositions and conjunctions and bear slots to be filled, for instance, “当…的时候 (by the time…).” Sentence builders differ with phrasal constraints in terms of syntactic levels (Wang, 2013, p. 46). Moreover, institutionalized expressions were first proposed by Nattinger and DeCarrico (1992, p. 45), which include “proverbs, aphorisms, and formulas for social interaction” and all other chunks efficient for a speaker to “store as units.” Wang (2013, p. 45) raised that Mandarin institutionalized expressions include idioms with meaning inherited from ancient times and often entail a story or an allusion and conventional expressions are multiword phrases due to a long time of language use. He also included proper nouns, pragmatic markers, and conversational routines into consideration. Pragmatic markers in this study refer to multiword sequences that signal speakers’ communicative intentions and are distinct from the proposition content (Fraser, 1996). By contrast, conversation routines are transparent phrases that convey interpersonal functions, such as blessings, greetings, and apologies, etc. (Wang, 2013, p. 47).

**Table 1:** Taxonomy of Mandarin chunks for chunk identification.

Major chunk categories	Subordinate chunk categories	Examples
Collocation	Fixed collocation	一丁点儿 (a wee bit); 老朋友 (old friend); 酸甜苦辣 (sweets and bitters)
	Direct collocation	共商国事 (discuss state affairs together); 绝大多数 (overwhelming majority)
	Modified collocation	尽 (最大的) 努力 (try one’s (best) effort); 引起 (强烈的) 共鸣 (cause (strong) resonance)
Frame construction	Phrasal constraint	当 … 的时候 (by the time…); 自… 以来 (ever since…)
	Sentence builder	虽然 …但是 … (Even though…); 不但…而且… (Not only…but also)
Institutionalized expression	Idiom/Proverb/Allegorical saying	车到山前必有路 (things will eventually sort themselves out); 四面楚歌 (be besieged on all sides)
	Conventional expression	说心里话 (speak from the heart); 吃食堂 (eat in the dining hall)
	Pragmatic marker	你看 (you see); 我觉得 (I think);
	Proper noun	双城记 (a tale of two cities); 中华人民共和国 (People’s Republic of China)
	Conversational routine	对不起 (One’ apologies); 新年快乐 (Happy new year)

The validity of the identification was justified in two ways. Firstly, in previous studies, finalized chunks were the agreement by the majority of the judges. For instance, the thresholds were set between 66.7% (at least 2 out of 3 judges) by Wood (2006) to 71.43% (at least 5 out of 7 judges) by Foster (2001). As the number of judges doubled compared to Wood (2006), it was safe to set the agreement by at least 4 out of 6 participants as the minimum threshold for the current study. The average agreement score for each chunk category was from 85.24 to 98.55%, which was high above the minimum threshold in literature, indicating judges’ relatively high consensus in relation to specific chunk categories. Secondly, there were no significant differences between the number of chunks from each chunk category by each judge to the finalized results (*p* > 0.05, by the 2*10 chi-square test), indicating judges were following similar identification criteria in the task. Altogether, 1,149 chunk tokens were identified, including 462 collocations, 348 frame constructions, and 339 institutionalized expressions. The number of characters of the identified chunks is 3,540 and the related utterance reaches about 12.2 thousand characters. Utterances that were excluded from analysis is 19.9 thousand characters.

### 3.4. Data analysis

**Figure 1:**
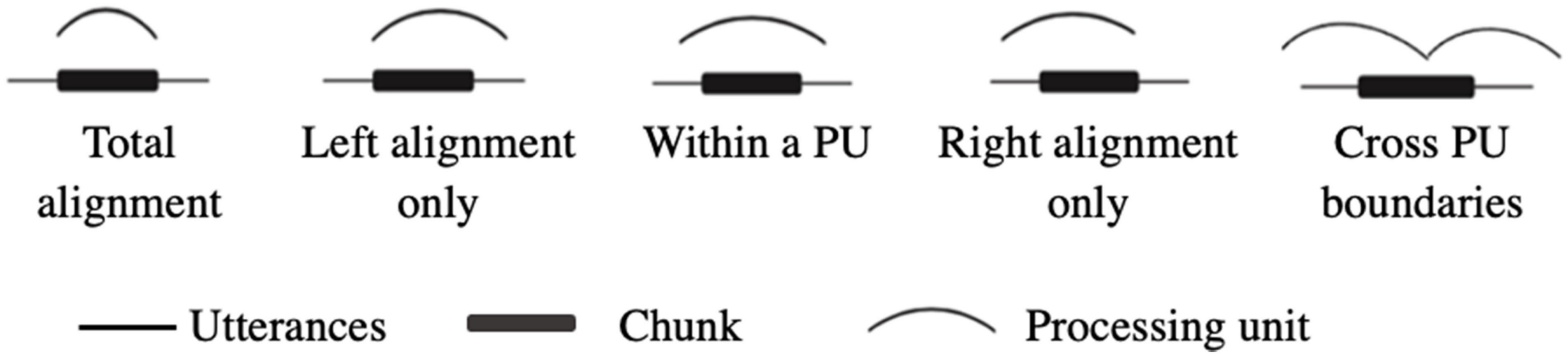
Situations of co-occurrence of chunks and processing units.

To address the first question, the study assessed the co-occurrence of chunks and processing units. This study adopted Lin and Adolphs’ (2009) proposal on the boundary alignment cases between chunks and intonation units, which included total boundary alignment, one-sided alignment on either the left or right side of chunks, and chunks totally embedded within a processing unit (shown in Figure 1). In addition, the current study added another situation where a chunk crosses boundaries of processing units, as pauses would possibly occur within a chunk. Distributions of chunks’ co-occurrence with processing units were evaluated by percentages.

The fluency of chunk production was assessed by pause placement before and in the middle of chunks and was also calculated by percentages. Chunks produced with grammatical pauses or without any pauses were regarded as fluent production of chunks, and chunks interrupted by hesitations were treated as chunk production with mental processing difficulties. In addition, hesitations before and in the middle of chunks were evaluated by their relative location to intonation units to explore how speakers encoded chunks with processing difficulties intonationally. Moreover, genre influence was assessed by comparing the co-occurrence of chunks and processing units between the formal and informal speech genres.

We adopted the chi-square test for homogeneity to evaluate the relationship between chunk categories and their co-occurrence with processing units since the data involved were discrete numerical data of the frequencies of co-occurrent situations and pause numbers. The number of chunks under each co-occurrence situation was first calculated and then applied to the chi-square function in R-studio. The same method was applied to assess the relationship between chunk categories and hesitation placement before and in the middle of chunks and the genre differences on chunk-PU co-occurrence as well. The study also did qualitative analyses of hesitation placement in relation to intonation units.

## 4. Results

RQ1: *Are chunks always delineated by pauses in mandarin spontaneous speech?*

By assessing the relationship between chunks and processing units (PU), the study found that pauses did not always mark the boundaries of chunks in Mandarin spontaneous speech. As shown in Table 2, overall, chunks that totally aligned with processing units made up 16.10%. Chunks that were contained within a processing unit were 69.67% (15.50% + 25.63% + 28.48). In addition, 14.29% of all chunks crossed the boundaries of processing units, indicating the involvement of pauses during their production.

**Table 2:** Co-occurrence of chunks and processing units.

	Total alignment	Left alignment only	Within a PU	Right alignment only	Cross PU boundaries
Total	16.10	15.50	25.63	28.48	14.29

**Figure 2:**
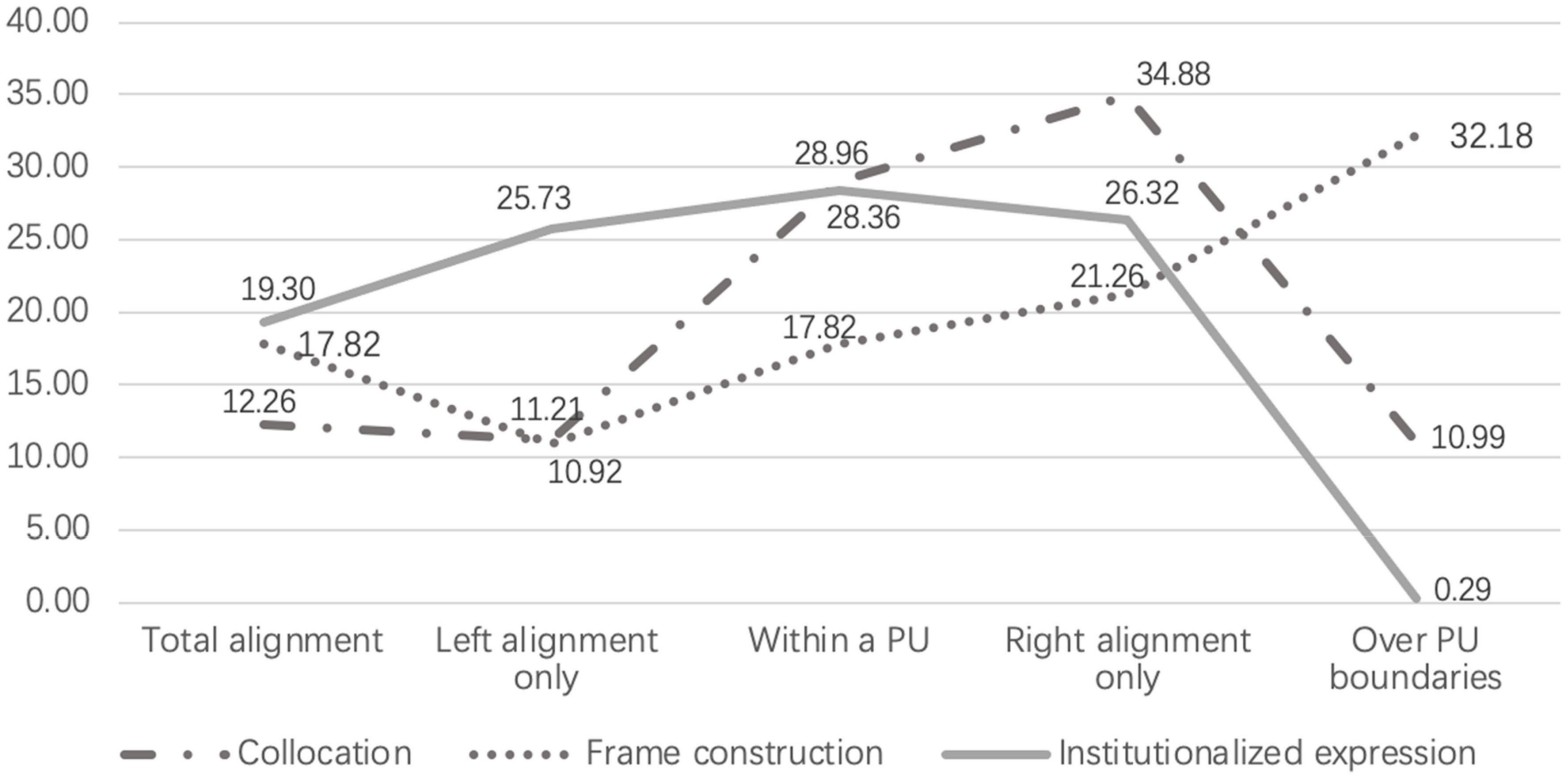
Co-occurrence of chunk categories and processing units.

The results further show that major chunk categories co-occur with processing units in different patterns. As Figure 2 illustrates, institutionalized expressions and frame constructions showed a similar possibility of occupying an independent processing unit by 19.30 and 17.82%, while only 12.26% of collocations totally aligned within processing units. Compared to total alignment with processing units, collocations were more likely to be situated by the right end of a processing unit by 34.88%, and institutionalized expressions tended to be situated within processing units by around 25.73 to 28.96%. In addition, major chunk categories differ in the possibility of crossing the boundaries of processing units. Frame constructions showed the highest possibility of spanning over a processing unit by 32.18%. 10.99% of collocations went over processing unit boundaries, and institutional expressions had the lowest probability of involving pauses during their production by 0.29%. Chi-square testing showed that major chunk categories co-occurred with processing units in significantly different patterns (*χ*^2^ = 194.397, *p* < 0.001).

RQ2: *Are chunks fluently processed in mandarin spontaneous speech?*

Overall, 83.07% of chunks were produced in a fluent way without any hesitation markers before and during production. Chunks with only hesitations ahead made up 5.82%, and with only internal hesitations reached 9.11%. Chunks with hesitations at both locations were 2.00%.

Table 3 illustrates the distribution of pausing situations before and in the middle of chunks. Chunks produced with a hesitation marker in front took up 7.98%, and chunks with internal hesitations showed a higher possibility of 11.18%. In addition, chunks tended to be fluently produced without any pauses involved, and grammatical pauses were more likely to be found before chunk production by 32.75%.

**Table 3:** Distribution of pause placemen and chunks.

	Fluent production		Hesitation
	Continuous	Grammatical pause	
Before	59.27	32.75	7.98
Middle	85.36	3.47	11.18

Major chunk categories exhibited variations in pause placement before chunk production. As shown in Table 4, collocations were the most likely to be produced without any pause markers ahead, and frame constructions were likely to encounter grammatical pauses. Chi-square testing showed that major chunk categories displayed significantly different patterns in terms of pause placement (*χ*^2^ = 43.544, p < 0.00 1). However, major chunk categories shared a similar probability of encountering hesitations in front by around 8%, and no significant difference was found in hesitation placement by the 2*3 chi-square test (*χ*^2^ = 0.385, *p* = 0.825).

**Table 4:** Pauses before chunks and chunk categories.

	Fluent production		Hesitation
	Continuous	Grammatical pause	
Collocations	69.74	21.89	8.39
Frame constructions	49.86	41.55	8.60
Institutionalized expressions	54.57	38.05	7.37

Moreover, major chunk categories showed varied patterns of internal pause placement in Mandarin spontaneous speech production (Table 5). Both collocations and institutionalized expressions shared a high probability of continuous production without any pauses. Comparatively, frame constructions were more likely to be produced with internal grammatical pauses. In addition, all institutionalized expressions were produced fluently. Internal hesitations were mainly found in collocations and frame constructions, and frame constructions had the highest probability of encountering internal processing difficulties. Chi-square testing revealed a significant difference among major chunk categories in internal pause placement (*χ*^2^ = 157.130, *p* < 0.001) and encountering internal hesitations (*χ*^2^ = 114.576, *p* < 0.001).

**Table 5:** Pauses in the middle of chunks and chunk categories.

	Fluent production		Hesitation
	Continuous	Grammatical pause	
Collocations	88.84	2.36	8.80
Frame constructions	66.76	8.02	25.21
Institutionalized expressions	99.71	0.29	0.00

Hesitations around chunks showed different tendencies in co-occurring with the boundaries of intonation units. As shown in Table 6, hesitations before chunk production were more likely to be found at the boundaries of intonation units. In contrast, internal hesitations of chunks showed a higher probability of being produced within an intonation unit, indicating speakers tended to encode chunks with processing difficulties with a coherent intonation contour.

**Table 6:** Hesitation placement in relation to intonation units.

	At boundaries of intonation units	Within intonation units
Before	81.52	18.48
Middle	53.47	46.53

RQ3: *Do chunks co-occur with processing units in a similar way across speech genres?*

The formal and informal speech data showed varied patterns of co-occurrence of chunks and processing units. As Table 7 illustrates, the formal speech contained more chunks spanning over the boundaries of processing units and fewer chunks occupying an independent processing unit. In Contrast, chunks in the informal speech were more likely to be realized by a single processing unit and to be produced within one processing unit. In addition, the informal speech had more chunks that aligned the left boundaries of processing units than formal speech. The two types of speech genres shared a similar threshold of chunks aligning the right boundaries of processing units. Chi-square testing showed that the formal and informal speech differ significantly in the co-occurrence patterns of chunks and processing units (*χ*^2^ = 37.817, *p* < 0.001).

**Table 7:** Co-occurrence of chunks and processing units in formal and informal speech data.

	Total alignment	Left alignment only	Within a PU	Right alignment only	Cross PU boundaries
Formal	9.39	6.57	31.92	37.09	15.02
Informal	15.44	15.06	27.41	33.98	8.11

The influence of speech genre on the mental processing of chunks has also been found in each major chunk category (see Table 8). Collocations were more likely to totally align with processing units in informal speech and cross the boundaries of processing units in formal speech. Chi-square testing showed collocations co-occurred with processing units in significantly different patterns in formal and informal speech settings (*χ*^2^ = 16.970, *p* = 0.002). In addition, frame constructions shared a similar threshold of occupying more than one processing unit, but they were more likely to align both processing unit boundaries in informal speech. According to the chi-square testing results, co-occurrence patterns of frame constructions and processing units were significantly different between formal and informal speech settings (*χ*^2^ = 11.530, *p* = 0.021). Moreover, institutionalized expressions in informal speech showed a much higher tendency to co-occur with processing units totally. Chi-square testing supported a significant difference between the formal and informal speech in the distributional patterns of institutionalized expressions and processing units (*χ*^2^ = 33.888, *p* < 0.001).

**Table 8:** Co-occurrence of chunk categories and processing units in formal and informal speech.

Chunk category	Speech genre	Total alignment	Left alignment only	Within a PU	Right alignment only	Cross PU boundaries
Collocations	Formal	9.39	6.57	31.92	37.09	15.02
	Informal	15.44	15.06	27.41	33.98	8.11
Frame constructions	Formal	13.72	9.29	19.91	23.89	33.19
	Informal	25.41	13.93	13.93	16.39	30.33
Institutionalized expressions	Formal	8.65	29.73	35.14	26.49	0.00
	Informal	31.85	21.02	20.38	26.11	0.64

## 5. Discussion

### 5.1. Chunks and processing units

The findings of this study support the tendency of holistic processing of chunks in spontaneous speech, as the majority of chunks were produced within pause-defined units. Chunks have been long suggested to be holistically processed due to holistic storage and retrieval (Wray, 2002), contributing to its prosodic manifestation in the way of less involving internal pauses. Compared to Lin’s (2018) results of 82.26% of 62 chunks in adult English speech production, this study has a lower ratio of chunks within a pause-defined unit due to a larger number of chunks and more chunk types in the calculation. In addition, the study also considered multiple hesitation phenomena into analysis, for instance, filled pause and drawls, which also contribute to the decrease of probability of chunks situated within pause-defined units. However, most chunks were produced within a pause-defined unit, indicating the tendency for holistic processing of chunks by speakers in spontaneous speech.

In this study, the co-occurrence of chunks and pause-defined units was significantly influenced by chunk categories, indicating different mental processes during chunk production. As previous studies suggested, collocations, binomials, and idioms undergo different psychological processes regarding their specific properties, such as compositionality, syntactic level, and phrase types (Carrol and Conklin, 2019). Chunk properties, such as fixedness, continuity, meaning transparency, and grammatical level, are on a continuum (Nattinger and DeCarrico, 1992; Wray, 2002) and influence the prosodic realization of chunks. In the current study, institutionalized expressions, including meaning opaque idioms, conventionalized expressions, and highly functional pragmatic markers and conversational routines, were more likely to be realized by one processing unit and resisted internal hesitations. In contrast, frame constructions that are discontinuous with open slots to be filled were found more likely to cross the boundaries of pause-defined units, indicating more cognitive effort in speech planning in discontinuous chunks on the phrasal and sentential levels. In addition, collocations consisting of highly fixed and semi-fixed restricted forms showed the probability of crossing the pause-defined unit boundaries between frame constructions and institutionalized expressions. Regarding the tendencies of frame constructions and collocations in crossing the boundaries of processing units, it can be deduced that analytical processes were involved during the production of these types of chunks.

Moreover, chunks tend to be situated within processing units instead of total alignment. The low ratio of total alignment between chunks and processing units supports Bardovi-Harlig’s (2009) observation that native speakers continue to talk after chunks, without pauses. Apart from 14.29% of chunks spanning over boundaries of pause-defined units, most chunks were produced within one pause-defined unit, which has also been reported in Dahlmann and Adolphs (2007) and Lin (2018). This supports that chunks can provide “short-cuts” in speech planning and are “time-buyers” for language users to promote speech fluency (Wray and Perkins, 2000, p. 16), enabling speakers to process more information in one processing unit other than one chunk in a planning unit in spontaneous speech. A processing unit can consist of more than one storage unit, and the alignment between storage units and holistic units depends on the information required in the context (Lin, 2018, p. 49). Despite the holistic processing of chunks, what is holistically processed is yet to be revealed. In addition, none of the chunk categories exhibit a high level of total co-occurrence with pause-defined units, not supporting chunks as processing units in spontaneous speech production, as suggested in previous studies (Myles and Cordier, 2017). According to Tao’s (1996) proposal based on the grammatical analysis of intonation units, speech units in Mandarin conversations mainly consist of nominal phrases, verb expressions, and argument-verb combinations. However, in spontaneous monologues, collocations, including noun combinations, and verb phrases, did not show a high level of co-occurrence with paused-defined units. The processing units of Mandarin monologs are worthy of further discussion.

### 5.2. Chunks and pause placement

The findings on chunks and pause placement support the cognitive fluency of chunk production at both stages of retrieval and production. Junctures before chunks would indicate mental retrieval or speech planning before chunk production, and those in the middle of chunks would indicate the mental processing process during chunk production. Psycholinguistic studies on holistic storage and processing support the mental processing advantages of chunks over novel strings (*cf.* Underwood et al., 2004; Kim and Kim, 2012). This study has found 83.07% of chunks free of processing difficulties at both retrieval and production stages and only 2.00% of chunks with processing difficulties at both stages, supporting the cognitive fluency for chunk production in spontaneous speech production (Erman, 2007).

Different patterns of pause placement also support the influence of chunk properties on mental processing before and in the middle of chunks. Weinert (1995) proposed that a frame with a fillable slot could be retrieved less holistically than continuous strings or idiomatic sequences, for it involves additional processing effort of lexical searching grammatically and contextually. According to the current findings, frame constructions are the most likely to involve grammatical pauses and hesitations, revealing additional processing efforts for lexical searching to fill the open slots. In addition, frame constructions, such as sentence builders, require speakers not only to produce chunks but also to organize the utterance grammatically. On the other hand, collocations showed a lower probability of encountering hesitations and grammatical pauses, as collocations consisted of both semi-fixed and fixed forms and required less processing effort than frame constructions. In contrast, highly fixed institutionalized expressions were all produced without internal hesitations. The current findings correspond to Erman’s (2007) conclusion that cognitive fluency is influenced by the degree of fixedness of prefabricated sequences. However, though sharing fundamental differences in structural and functional properties, chunks of different categories showed no significant differences in encountering hesitations before the production of chunks, indicating shared cognitive fluency at the stage of chunk retrieval across chunk categories.

### 5.3. Hesitation placement and phonological coherence

Speakers’ tendency to maintain phonological coherence on chunk production when encountering processing difficulties supports the holistic processing of chunks. As previous studies suggested, chunks tend to be produced under a continuous contour due to holistic storage and processing (Wood, 2006; Lin and Adolphs, 2009; Lin, 2018). However, this tendency cannot be equalized to the cognitive fluency of chunk production, as intonation units may involve hesitations inside. The findings showed that speakers did encounter processing difficulties during chunk production, even when they produced chunks in one intonation unit. On the one hand, hesitations before chunk production were more likely to co-occur with intonation unit boundaries, which correspond to previous findings on hesitation placement with intonation units (Boomer, 1965; Clark and Tree, 2002).

**Figure 3:**
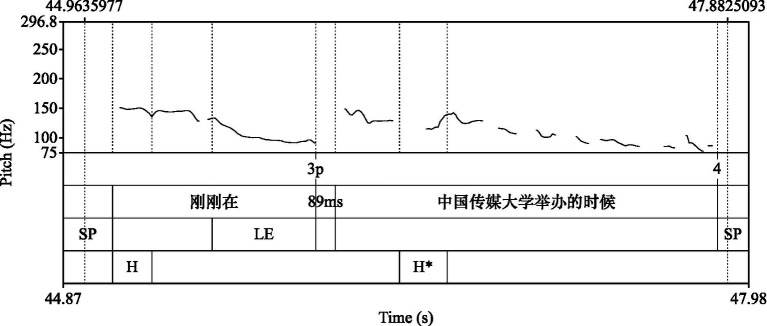
A drawl in the phrasal constraints “在…的时候 by the time when…).”

On the other hand, hesitations in the middle of chunks showed a stronger tendency to be produced within intonation units. According to Clark and Tree (2002, p. 97), the “local importance” or disruptiveness of silent pauses within an intonation unit is greater, and speakers tend to realize the pause with a pause filler. Suppose chunks are holistic units restored in mental speakers’ mental lexicon. In that case, speakers will avoid silent pauses to disrupt the holistic structure of chunks. Figure 3 shows a typical case where the speaker encountered information search difficulties in the production of the phrasal constraint “在…的时候 (by the time when…).” Instead of employing silent pauses, the speaker lengthened the syllable of “在,” creating a drawl to buy more time for organizing the filled information. In this way, the speaker maintained the phrasal constraint within a coherent intonation contour and manifested the phonological coherence of chunks (Lin, 2018). As suggested by Chafe (1994), a coherent intonation contour represents a single focus of consciousness and the chunkiness of information. The tendency that speakers maintain chunks within an intonation unit when they encounter processing difficulties during the production of chunks is regarded as evidence of holistic processing of chunks.

### 5.4. Genre difference on chunk processing

The current findings support that the mental processing of chunks could be stylistically different regarding different degrees of speech formality and speech topics. Erman (2007) suggested that the lexical choices for fixed and semi-fixed slots in chunks were confined by the speech context, and different speaker groups might store different chunks in their mental lexicon. In the current study, the formal speech data contained more chunks crossing the boundaries of processing units, indicating more cognitive efforts in chunk production under the formal speech setting. On the one hand, the formal speech was done through live broadcasting, and the topics required sensitive and accurate political comments, which increased the psychological pressure during speech production. On the other hand, speakers in formal settings were researchers who were less competent in handling complex speech situations than the well-trained hosts and hostesses. Moreover, the genre influence on the mental processing of chunks was found in each major chunk category. However, major chunk categories exhibited similar tendencies of crossing the boundaries of processing units. For instance, frame constructions in both speech settings were the most likely to occupy more than one processing unit, and nearly all institutionalized expressions were produced by one processing unit. This indicates that the properties of chunks, such as fixedness and continuity, influence the processing of chunks across speech genres.

## 6. Conclusion

The primary aim of this study was to examine whether chunks are marked by pauses and performed as processing units in processing units in Mandarin spontaneous speech. We examined the co-occurrence of chunks and pause-defined processing units and the influence of chunk categories with fundamental differences in structural and functional properties on such co-occurrence. Our results support the tendency of holistic processing and the significant influence of chunks’ formal and functional properties on their co-occurrence with processing units. The secondary aim of this study was to examine to what extent chunks would encounter hesitations and whether hesitations for chunk production would interrupt the intonational continuity of chunks. Our results support that most chunks resist hesitations before and during chunk production, indicating cognitive fluency of chunks at both retrieval and production stages. Major chunk categories shared significantly different patterns of pause placement before and during chunk production. Which also reveals different mental processes for the production of different chunk categories. Our results further revealed that speakers tended to maintain the phonological coherence of chunks, revealing the mental holistic nature of chunks. Thirdly, the mental processing of chunks can be stylistically different due to the degree of speech formality and discourse topics, as chunks co-occur with processing units in significant patterns between the formal and informal speech genres. In addition, the genre influence over chunk processing has also been found in each major chunk category.

The current study has provided empirical evidence for chunks to be holistically processed in spontaneous speech due to holistic storage and retrieval, as proposed by Wray (2002). Despite the tendency of holistic processing, the study showed that analytical processes also occur in the production of frame constructions and collocations, as both grammatical pauses and hesitations were located during the preparation and production of these chunks. Moreover, our findings further the understanding of the phonological coherence of chunks. On the one hand, it showed the tendency of chunks to be produced by one intonation unit, as hesitations before chunks were likely to align with intonation unit boundaries. On the other hand, phonological coherence is not necessarily equal to the cognitive fluency of chunks, as chunks produced by one intonation unit could involve processing difficulties. Additionally, the current study provided pedagogical NLP insight into the spontaneous realization of chunks, as it revealed how native speakers compensate for processing difficulties through hesitations. It should also be reminded that although chunks enjoy a high level of holistic processing, they do not have to be processing units in speech production. The role that chunks play in speech production is supportive, as they provide ease for speakers to involve more information in one processing unit in spontaneous speech.

The limitations of the current study lie in two main perspectives. Firstly, the discussion of the influence of speech genre in this study could have been bold and over-generalized. The components of a genre encompass multiple perspectives, including speech topics, settings, and ways of speech delivery, which facilitate an intrinsic impact on speech processing and speech production. Future analysis of genre influence would decompose genre into more specific factors in the speech setting. In addition, future research could add more diversity to speech types such as teacher’s lectures, presidential speeches, interviews, and conversations. Secondly, though we have reported data that support the significant influence of chunk properties and genre factors on the mental processing of chunks, it remains further statistical efforts on the different tendencies of each comparison.

## Data availability statement

The raw data supporting the conclusions of this article will be made available by the authors, without undue reservation.

## Author contributions

DX designed the study, performed the data collection and analysis, and wrote and edited the manuscript. HC and BL supervised the research. HC provided suggestions for the research design, organized data collection, and reviewed the manuscript. BL revised the organization and wording of the manuscript and reviewed data analysis and presentation. All authors contributed to the article and approved the submitted version.

## Funding

This work was supported by the National Social Science Foundation of China [grant number 20AYY013].

## Conflict of interest

The authors declare that the research was conducted in the absence of any commercial or financial relationships that could be construed as a potential conflict of interest.

## Publisher’s note

All claims expressed in this article are solely those of the authors and do not necessarily represent those of their affiliated organizations, or those of the publisher, the editors and the reviewers. Any product that may be evaluated in this article, or claim that may be made by its manufacturer, is not guaranteed or endorsed by the publisher.

## Footnotes

^1^COLT is the short name for the Bergen Corpus of London Teenager Language, and LLC is short for the London-Lund Corpus of spoken English (Erman, 2007).

^2^The Switchboard NXT Corpus contains telephone conversations between unacquainted adults (Schneider, 2014). NXT is related to two transcription methods, including the Treebank3 and the MS-state transcript.

^3^NMMC is the short name for the Nottingham Multimodal Corpus (Lin, 2018).

^4^NICLEs-CHN is the short name for the Nottingham International Corpus of Learner English (spoken)-Chinese learner sub-corpus (Lin, 2018).

^5^The formal speech data is the selection of the open source of TV broadcasting program Across the Straits (https://tv.cctv.com/lm/hxla/?spm=C52507945305.Pg6GRYzEVgqy.0.0).

^6^The informal speech data is the selection from the open source of the host speech at the closing ceremony of the First International Film Festival (https://v.youku.com/v_show/id_XMzA0MzY1MTYwMA==.html).
